# Pain neuroscience education improves knowledge and satisfaction in adolescents with and without intellectual disabilities: a cross-sectional study

**DOI:** 10.1017/neu.2025.10041

**Published:** 2025-10-30

**Authors:** Carlos Fernández-Morales, María de los Ángeles Cardero-Durán, Luis Espejo-Antúnez

**Affiliations:** Department of Medical-Surgical Therapy, Faculty of Medicine and Health Sciences, University of Extremadura, Badajoz, Spain

**Keywords:** pain neuroscience education, intellectual disability, school health, adolescents, inclusive education

## Abstract

To assess satisfaction and pain-related knowledge levels following an inclusive Pain Neuroscience Education (PNE) programme in improving pain-related knowledge and perceived satisfaction among adolescents with and without intellectual disabilities, and to assess its applicability in digital health education settings. Methods: A multicentre, cross-sectional study was conducted in 15 public schools. A total of 373 students (5th–6th grade), including those with intellectual disabilities, participated in a hybrid-format PNE programme delivered in two 90-minute sessions. Satisfaction and knowledge were assessed using an adapted, easy-to-read questionnaire, with exploratory factor analysis identifying three core domains: activity format, teacher evaluation, and SDG-related training. Results: Overall satisfaction and knowledge gains were high across all participants. No significant differences were found between students with and without intellectual disabilities or between urban and rural schools in satisfaction and teacher evaluation. However, rural students reported greater awareness of the SDG-related content (p < 0.05). Conclusion: The adapted PNE programme was well-received and associated with high levels of pain-related knowledge across diverse educational contexts. Its inclusive and hybrid design supports its potential scalability through digital health strategies, promoting equity in pain education.

## Summation

- An inclusive, school-based pain neuroscience education (PNE) programme significantly improved pain-related knowledge and satisfaction in adolescents with and without intellectual disabilities.
- The intervention was equally effective across rural and urban settings and between students from mainstream and special education centres.
- The hybrid format and use of easy-to-read materials support the programme’s feasibility as a scalable digital health strategy to promote health literacy in diverse educational populations.

## Considerations

- The cross-sectional design limits causal interpretations and prevents assessment of long-term retention of knowledge or behavioural changes.
- The study sample was restricted to a single geographical region, potentially limiting generalisability to other educational or cultural contexts.
- Although the questionnaire showed good internal consistency, further psychometric validation is warranted to confirm its construct validity across populations with intellectual disabilities.

## Introduction

Spinal pain is a highly prevalent musculoskeletal condition among adolescents, with estimates ranging from 18.2% at age 10 to 65.6% by age 16 (Calvo-Muñoz *et al*., 2013). In Spain, recent data place the prevalence at 34.3% in school-aged populations (Fraiz Barbeito *et al*., 2021). The impact of adolescent pain extends beyond physical symptoms, contributing to reduced participation in daily activities, emotional distress, and poorer health-related quality of life (Pellisé et al., 2009; Millera *et al*., 2018). Previous studies have shown that adolescents with persistent pain often report poorer health-related quality of life and lower self-esteem or self-efficacy (Mikkelsen *et al*., 2021), and that chronic pain in adolescence is associated with higher levels of anxiety and internalising symptoms (Noel *et al*., 2016; Dudeney *et al*., 2023). These issues may be further exacerbated in students with intellectual disabilities, who often experience barriers in communicating pain and accessing health-related education (Weissman-Fogel *et al*., 2015; Genik *et al*., 2017).

Despite its relevance, pain is rarely addressed systematically in primary education settings. There is growing consensus on the need to promote pain literacy from an early age to foster healthy beliefs, encourage self-management, and reduce long-term burden (Galmés-Panadés *et al*., 2023). PNE is one such approach. PNE is an educational strategy that explains basic pain neurobiology, helps to reframe unhelpful beliefs, and promotes adaptive coping (Louw *et al*., 2018). It has shown promising results in challenging unhelpful beliefs and promoting active coping strategies in adults and children with musculoskeletal pain (Louw *et al*., 2018; Wood & Hendrick, 2019). Although increasingly integrated into clinical contexts, its use in school-based settings, especially for students with special needs, remains underexplored.

Recent calls have emphasised the role of inclusive health education in promoting equity and reducing disparities (Genik *et al*., 2017; Zamora-Polo & Sánchez-Martín, 2019). In alignment with the Sustainable Development Goals (General Assembly of the United Nations, 2015), educational systems are expected to address topics such as well-being, inclusion, and sustainability. However, children with intellectual disabilities often remain excluded from mainstream health promotion initiatives (Fajardo *et al*., 2014), partly due to a lack of accessible formats and trained staff.

Technological advances and the growing use of digital health tools offer an opportunity to bridge these gaps (Krupinski & Weinstein, 2013). When adapted appropriately, hybrid learning formats may increase the reach of pain education across diverse school populations, including students in rural or underserved areas (Pérez-Foguet *et al*., 2018; Fisher & Louw, 2022). At the institutional level, effective collaboration between educational centres and health professionals is essential to implement scalable and impactful interventions (Gross *et al*., 2012).

The present study aims to evaluate the effectiveness of an inclusive, school-based PNE programme in improving pain knowledge and satisfaction in adolescents with and without intellectual disabilities. The study also explores differences across urban and rural schools and considers the potential applicability of this educational model in digital health contexts.

## Material and methods

### Design

A multicentre, observational, descriptive cross-sectional study was carried out in 15 academic centres across the Autonomous Community of Extremadura (Spain). The study was prospectively registered at ClinicalTrials.gov (NCT06664606) and approved by the Bioethics Committee of the University of Extremadura (approval number 112/2024). It was conducted in accordance with the *Strengthening the Reporting of Observational Studies in Epidemiology* (STROBE) guidelines (von Elm *et al*., 2008). Additionally, the domains of structural validity and internal consistency were assessed following the COSMIN criteria for health measurement instruments (Mokkink *et al*., 2016).

### Participants

Participants were fifth- and sixth-grade primary school students from the Autonomous Community of Extremadura (Spain), chosen by convenience sampling. Educational centres were divided geographically into rural centres, defined as those located in municipalities with a population of less than 10.000 inhabitants, and urban centres. Additionally, the sample included a classification between special education centres, comprising students with intellectual disabilities, and ordinary education centres. Written informed consent was obtained from parents or legal guardians, and assent was obtained from all participating students.

The inclusion criteria were (i) students belonging to the network of educational centres of preferential attention designated by the Department of Education of the Regional Government of Extremadura in the 2020-21 academic year and (ii) students enrolled in the 5th and 6th grades of primary education. The exclusion criteria were (i) students who did not regularly attend the academic activities of the course, (ii) those who did not attend both sessions and complete the questionnaire, and (iii) students diagnosed with chronic pain of an oncological nature.

**Figure 1.**
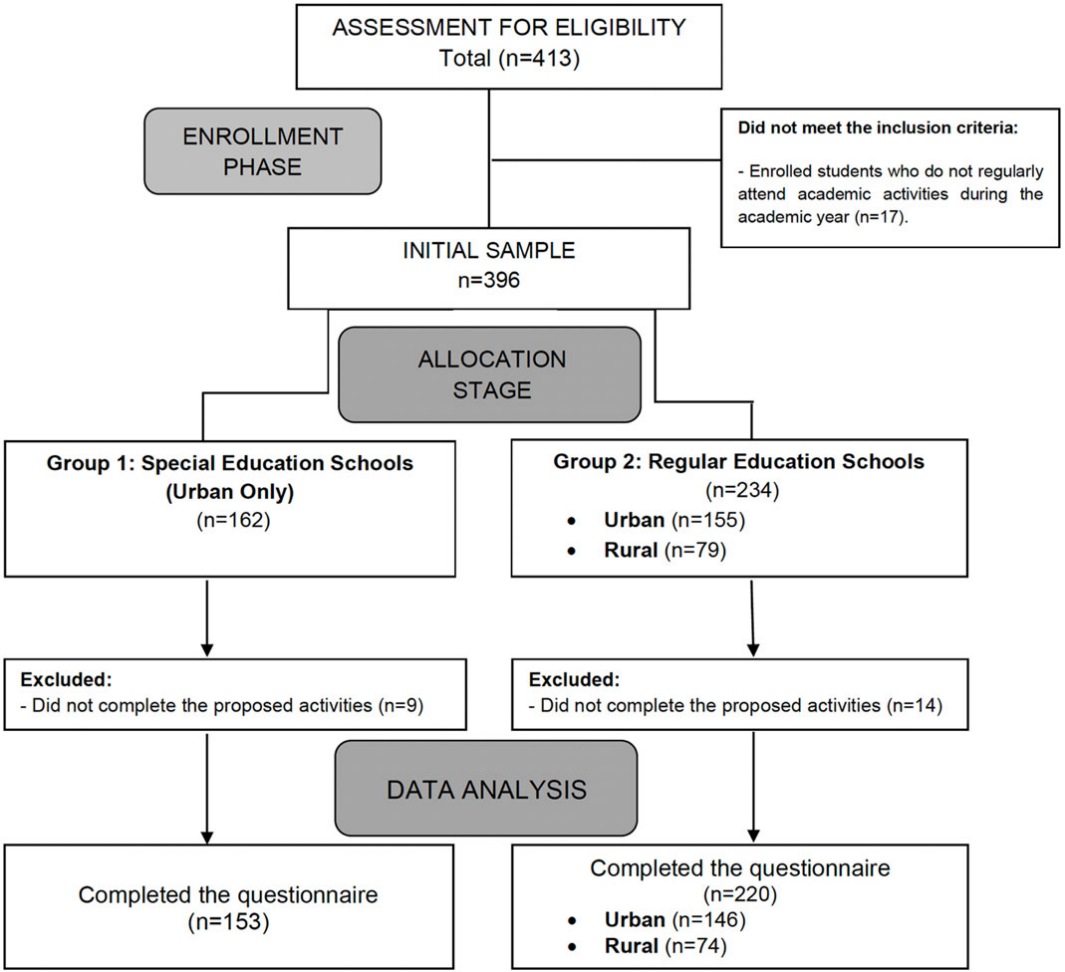
Flow diagram of the participants throughout the study.

Of the 413 potentially eligible participants, a total of 396 students formed the initial sample after applying the inclusion and exclusion criteria. Subsequently, 23 students were excluded because they did not fully complete the proposed activities, resulting in a final sample of 373 students. The recruitment period went from 1 October 2021 to 31 May 2022. A flow diagram of the participants throughout the study is provided in Fig. 1.

### Pain neuroscience education programme for students

**Figure 2.**
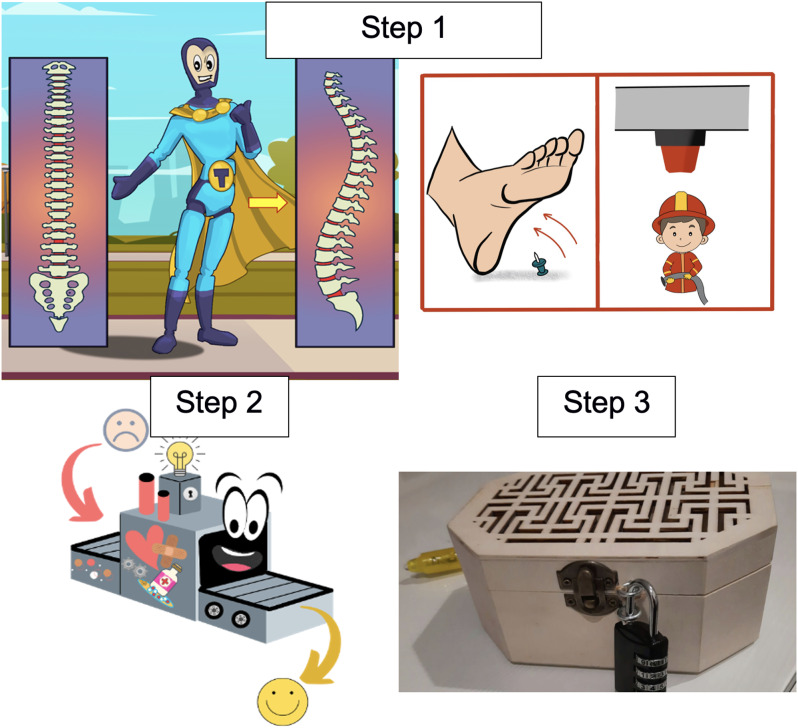
Examples of the educational activities developed: step 1) superhero explaining pain anatomy and neurophysiology concepts, and comics on pain analogies; step 2) “Pain repair” machine; step 3) escape room chest. Resources developed by the authors.

The PNE programme was implemented in each participating educational centre. The protocol design was based on previous PNE interventions conducted in academic settings (Louw *et al*., 2011; Louw *et al*., 2018; Saracoglu *et al*., 2021). Each session was delivered by the same physical therapist, who had extensive teaching experience in the design and implementation of PNE programmes. The content was organised into three steps, structured in a hybrid format. The materials for steps 1 and 2 were developed in digital format, whereas the content for step 3 was delivered in print (Fig. 2).

#### Step 1: introduction and theoretical-practical educational activity

A series of digital materials, including posters, brochures, and illustrative comics, were presented in a multimedia format to explain key concepts of pain anatomy and neurophysiology, as well as to demystify common misconceptions related to pain. Subsequently, students answered questions applied to everyday situations in a digital format (using true or false). The objective was to receive feedback on the level of attention during the class and to identify potential comprehension difficulties.

#### Step 2: group reflection activity using the “Pain repair machine”

This element, designed for the project, invited students to reflect on their beliefs about pain, construct new ideas, and reinforce active coping strategies in a collaborative context.

#### Step 3: educational escape room on pain

In this final activity, students had to solve a series of riddles and puzzles to discover pain-related keywords, which they then used to unlock a chest with a final reward. This step was designed to consolidate knowledge in a playful and collaborative way.

Two 90-minute sessions were conducted on separate days within the same week. The first session covered Step 1 of the programme (90 minutes). The second session began with a brief review of the previous content (15 minutes) and continued with Steps 2 (30 minutes) and 3 (45 minutes), covering the proposed activities for all students in each class.

### Questionnaire

The instrument used to assess participants’ satisfaction and their perception of the educational activity was a structured questionnaire. The questionnaire was specifically designed for this study and consists of 18 items assessing different aspects related to the educational activity. The questions cover topics such as the organisation, duration, and materials of the activity, as well as aspects related to the interest and participation of the students and the clarity in explaining the contents. Additionally, items assessing students’ level of knowledge and perception of the SDGs and their relation to the activity are included. The response scale for all items is a 7-point Likert-type scale, where 1 represents the lowest score and 7 the highest.

The questionnaire was adapted to facilitate its comprehension, ensuring its accessibility through an easy-to-read format, following the principles of cognitive adaptation developed by the Aexpainba Center (Badajoz) (Supplementary Material 1). It was administered anonymously by classroom teachers after the sessions, ensuring that responses could not be linked to individual students and minimising potential social desirability bias.

### Statistical analysis

Data analysis was conducted using SPSS version 26 (IBM Corp., Armonk, NY, USA). The normality of continuous variables was assessed using the Kolmogorov–Smirnov test, while the Chi-square goodness-of-fit test was applied to categorical variables. Descriptive statistics were calculated for each variable: continuous data are reported as mean ± standard deviation (SD), and categorical data as frequencies (%).

Independent *t*-tests were used to compare mean scores between groups (rural vs. urban schools; special vs. mainstream education centres). When the assumption of homogeneity of variances was violated, Welch’s *t*-test was applied. Associations between categorical variables were evaluated using the chi-square test of independence. Statistical significance was set at *p* < .05.

An exploratory factor analysis (EFA) was performed to examine the underlying structure of the data, using principal component analysis (PCA) for dimensionality reduction while retaining key variance (Osborne & Fitzpatrick, 2012). Sampling adequacy was assessed using the Kaiser–Meyer–Olkin (KMO) measure, with values above 0.50 considered acceptable. Bartlett’s test of sphericity was conducted to verify the presence of significant correlations among items. To facilitate interpretability, a Varimax orthogonal rotation was applied.

Internal consistency of the resulting factors was evaluated using Cronbach’s alpha and omega coefficients (Roter & Larson, 2002). Additionally, individual-level reliability was estimated by calculating the standard error of measurement (SEM) for each factor using the formula SEM = SD × √(1 – Cronbach’s α), as recommended by Beattie *et al*. (2007).

## Results

### Sample description

A total of 373 students from 15 schools in the region of Extremadura participated, of which 5 were rural public schools and 10 urban public schools, and 3 were special education schools and 12 regular education schools. Table 1 shows the demographic characteristics and school distribution of the participants. At baseline, no statistically significant differences were found between males and females regarding age, school location (urban vs. rural), or school type (special education vs. regular education).

**Table 1.** Demographic and school characteristics of the study sample

Characteristics	Mean (SD)/*n* (%)	Median	Minimum	Maximum
**Age (years)**	11.84 ± 0.9	11.79 ± 1.23	9	14
**Gender**				
**Male**	158 (42.4)			
**Female**	215 (57.6)			
**Course/Year**				
**5** ^**th**^	210 (56.3)			
**6** ^**th**^	163 (43.7)			
**School Location**				
**Urban Public Schools**	299 (80.2)			
**Rural Public Schools**	74 (19.8)			
**School Type**				
**Special Education Schools**	153 (41)			
**Regular Education Schools**	220 (59)			

Data are reported as mean ± SD for quantitative variable or frequency and (%) for qualitative outcomes. SD = Standard Deviation.

### Appropriateness: sampling adequacy and sphericity

The factor analysis of the 18 items showed a Kaiser–Meyer–Olkin measure of sampling adequacy of 0.836. Likewise, the Barlett test allowed us to reject the null hypothesis (sphericity) (*p* < 0.001).

### Factor structure of the questionnaire

The distribution of the variance explained by the factors extracted from the principal component analysis showed a structure of the questionnaire organised into three constructs, together explaining 61.51% of the accumulated variance. The first construct explains 39.47% of the variance, followed by the second construct with 11.55% and the third construct with 10.48%. The first construct groups questions related to “Activity Format”, the second construct is associated with “Teacher Evaluation,” and the third construct includes items on “Training based on the Sustainable Development Goals (SDGs)” (Table 2).

**Table 2.** Rotated component matrix*

	Component (Factors)		
Item	Activity Format	Teacher Evaluation	SDGs-Based Education
Q1. Knowledge of the SDGs	−0.064	−0.040	**0.808**
Q2. Perceived relation of the activity to the SDGs	−0.002	−0.018	**0.791**
Q3. Information on SDGs from media sources	−0.042	−0.054	**0.781**
Q4. Organisation of the activity	**0.757**	−0.001	0.024
Q5. Duration of the activity	**0.864**	0.076	−0.167
Q6. Timing of the activity	**0.950**	0.042	−0.094
Q7. Knowledge gained by students	**0.913**	−0.008	0.011
Q8. Explanation of campaign objectives	**0.003**	−0.038	−0.095
Q9. Ease of content	**0.822**	0.063	−0.144
Q10. Materials shown (e.g., plastic spine)	**0.956**	0.033	−0.034
Q11. PowerPoint presentation	**0.925**	0.050	−0.104
Q12. Achievement of the lecture’s objectives	**0.021**	−0.026	−0.066
Q13. General opinion on the lecture	**0.930**	0.043	−0.047
Q14. Recommendation of this activity to other schools	**0.792**	−0.017	−0.005
Q15. Teacher’s ability to maintain student interest	0.014	**0.677**	0.074
Q16. Appropriateness of teaching pace	0.089	**0.646**	0.051
Q17. Encouragement of student participation	−0.040	**0.755**	−0.006
Q18. Overall evaluation of the teacher	0.079	**0.771**	0.102

* Extraction method: principal component analysis. Rotation method: Varimax with Kaiser normalisation.

Bold: these factorial weights are put in bold to highlight which were the items that correspond to each factor.

### Internal consistency reliability of the questionnaire

In the reliability analysis, the questionnaire obtained a Cronbach’s alpha of 0.728 and an Omega coefficient of 0.704, indicating adequate internal consistency. The reliability and SEM results for each construct of the questionnaire are shown in Table 3.

**Table 3.** Mean, standard deviation, minimum and maximum values of each questionnaire item and metric properties of the three constructs

	Min	Max	Mean	SD	*α*	*ω*	SEM
**Activity format**			**6.57**	**0.89**	**0.93**	**0.95**	**0.24**
Organisation of the activity (Q4)	1	7	6.81	0.67			
Duration of the activity (Q5)	1	7	6.31	1.31			
Timing of the activity (Q6)	1	7	6.53	1			
Knowledge gained by students (Q7)	1	7	6.66	0.9			
Explanation of campaign objectives (Q8)	1	7	6.44	0.62			
Ease of content (Q9)	1	7	6.28	1.29			
Materials shown (e.g., plastic spine) (Q10)	1	7	6.61	0.92			
PowerPoint presentation (Q11)	1	7	6.44	1.25			
Achievement of the lecture’s objectives (Q12)	1	7	6.51	0.54			
General opinion on the lecture (Q13)	1	7	6.59	0.54			
Recommendation of this activity to other schools (Q14)	1	7	6.76	0.87			
**Teacher evaluation**			**6.58**	**0.74**	**0.68**	**0.68**	**0.42**
Teacher’s ability to maintain student interest (Q15)	1	7	6.66	0.93			
Appropriateness of teaching pace (Q16)	1	7	6.45	1.23			
Encouragement of student participation (Q17)	1	7	6.5	1.17			
Overall evaluation of the teacher (Q18)	1	7	6.72	0.81			
**SDG-based training**			**4.84**	**1.96**	**0.72**	**0.72**	**1.03**
Knowledge of the SDGs (Q1)	1	7	4.8	2.39			
Perceived relation of the activity to the SDGs (Q2)	1	7	5.5	2.21			
Information on SDGs from media sources (Q3)	1	7	4.6	2.48			

Min: Minimum, Max: Maximum, SD: Standard Deviation, α: Cronbach alpha, ω: Omega coefficient, SEM = Standard error of the measure.

### Descriptive data for the questionnaire

In the first construct on activity format, a mean score of 6.57 ± 0.89 was obtained; in the second construct on teacher evaluation, 6.58 ± 0.74; and in the third construct on SDG-based training, 4.84 ± 1.96. Tables 4 and 5 present the descriptive statistics of the responses obtained in the questionnaire by geographical distribution and type of school, respectively.

**Table 4.** Descriptive statistics of the sample and responses to the questionnaire between urban and rural public schools

	Urban Public Schools Group (*n* = 299)	Rural Public Schools Group (*n* = 74)	*p* Value *
**Age (years)**	11.82 ± 0.9	11.87 ± 0.9	0.72
**Gender**			
**Male**	130 (43.5)	28 (37.8)	0.38
**Female**	169 (56.5)	46 (62.5)	
**Course/Year**			
**5** ^**th**^	164 (54.8)	46 (62.2)	0.26
**6** ^**th**^	135 (45.2)	28 (37.8)	
**Activity format**	6.58 ± 0.89	6.51 ± 0.9	0.55
**Teacher evaluation**	6.56 ± 0.74	6.61 ± 0.78	0.62
**SDG-based training**	4.61 ± 2.05	5.77 ± 1.14	< 0.001*

Data are reported as mean ± SD for quantitative variables or frequency and (%) for qualitative outcomes. SD = Standard Deviation, SDG: Sustainable Development Goals. * Indicates between-groups statistical significance (*p* < 0.001).

**Table 5.** Descriptive statistics of the sample and responses to the questionnaire between special and regular education schools

	Special Education Schools Group (*n* = 153)	Regular Education Schools Group (*n* = 220)	*p* Value *
**Age (years)**	11.87 ± 0.9	11.77 ± 0.89	0.34
**Gender**			
**Male**	70 (45.8)	88 (40)	0.27
**Female**	83 (54.2)	132 (60)	
**Course/Year**			
**5** ^**th**^	95 (62.1)	115 (52.3)	0.06
**6** ^**th**^	58 (37.9)	105 (47.7)	
**Activity format**	6.5 ± 0.96	6.61 ± 0.84	0.8
**Teacher evaluation**	6.59 ± 0.74	6.56 ± 0.75	0.71
**SDG-based training**	5 ± 1.81	4.64 ± 2.13	0.14

Data are reported as mean ± SD for quantitative variables or frequency and (%) for qualitative outcomes. SD = Standard Deviation, SDG: Sustainable Development Goals. * Indicates between-groups statistical significance (*p* < 0.001).

In the comparison between rural and urban schools, no statistically significant differences (*p* > 0.05) were found in the constructs of activity format or teacher evaluation. However, in the construct related to the SDG-based training, a statistically significant higher score (*p* < 0.05) was observed among students from rural schools compared to those from urban schools (5.77 ± 1.14 vs. 4.61 ± 2.05).

In the comparison between Special Education Schools and Regular Education Schools, no statistically significant differences (*p* > 0.05) were found in any of the constructs analysed.

## Discussion

This study examined the level of satisfaction and pain knowledge acquired by primary school students – with and without intellectual disabilities – after participating in a PNE programme. Previous research has highlighted the potential of multi-stakeholder, school-based educational interventions to improve musculoskeletal health in adolescent populations (Fisher & Louw, 2022). In this context, pain education (PE) has been described as a *threshold concept*, capable of reshaping learners’ understanding of pain and encouraging more adaptive beliefs and behaviours (Smart, 2023). The identification of three factors in the questionnaire provides a practical framework for interpreting the students’ experience. Specifically, the domains reflect the format and organisation of the activities, the perceived role of the teacher, and the integration of content related to the Sustainable Development Goals (SDGs). Together, these domains capture the main elements that shaped students’ satisfaction and knowledge.

Integrating such programmes into eHealth platforms may enhance their accessibility and scalability within school environments. Digital formats offer the flexibility to tailor interventions to different cognitive levels and learning styles, including those of students with disabilities. For instance, gamified eHealth tools have demonstrated effectiveness in improving self-management of chronic conditions among young people (Maartje *et al*., 2024), suggesting that similar strategies could be beneficial for school-based pain education. In low-resource settings, the components of the programme (such as posters, comics, and escape-room activities) could also be implemented with print-only materials and simple classroom props, maintaining the same learning objectives (Kovacs *et al*., 2011; Fisher & Louw, 2022). When considering e-health scalability, common barriers such as limited connectivity in rural areas or insufficient teacher training should be acknowledged (Krupinski & Weinstein, 2013; World Health Organization, 2016), which may be mitigated through offline delivery formats, ready-to-use templates, and brief staff orientation.

Recently, Fisher and Louw (2022) have reported the benefits of uncomplicated, classroom-based interventions in low-resource settings. Among these interventions is PE, which has been shown to be effective in students across different educational stages (Saracoglu *et al*., 2021; Leysen *et al*., 2021), although it has not been investigated to date in adolescent students with and without intellectual disabilities. This may be due to the multiple and complex demands placed on special education schools. The findings of this study have allowed (1) to reduce inequalities in access to quality education and (2) to promote sustainable development in the field of health and well-being without placing an additional burden on teachers and parents, as the PNE programme intervention did not require leaving the classroom (General Assembly of the United Nations, 2015; Fisher & Louw, 2022).

The high satisfaction scores (6.43 ± 0.68) and the reported pain knowledge indicate that the proposed educational strategy appears to be effective. However, these results should be interpreted with caution, as the promotion of behavioural and belief change also requires other complementary initiatives, such as legislative strategies, healthy public policies, or social marketing (Gross *et al*., 2012). The observed improvements in knowledge levels align with the impact that education has had on pain-related attitudes and beliefs in previous studies conducted in academic settings (Saracoglu *et al*., 2021; Leysen *et al*., 2021; Fernández-Morales *et al*., 2025). Nevertheless, these results cannot be directly compared with those shown in the present study, as previous research has focused on adolescent students with neck pain and university students without intellectual disabilities (Leysen *et al*., 2021).

No statistically significant differences were found between rural and urban schools regarding the educational activity or teacher evaluation. However, a statistically significant difference was observed in the SDGs-related dimension, where students from rural schools scored higher than those in urban schools. On the other hand, the comparison between special education schools and regular schools did not show significant differences in any of the constructs analysed. Overall, the results show a fairly homogeneous perception of the programme among students with and without intellectual disabilities. The higher SDG scores in rural areas may reflect a closer connection with sustainability initiatives that are more visible in agricultural and community settings. At the same time, the absence of differences between special and mainstream schools reinforces the inclusive nature of the programme, as students with intellectual disabilities evaluated the activities in much the same way as their peers. It should also be noted that the teacher evaluation factor showed only modest internal consistency (*α* = 0.68). While acceptable for exploratory purposes, this suggests that further refinement and replication are needed in future studies.

The educational activity in pain neuroscience has proven to be effective in raising awareness and conveying key knowledge to students, regardless of their level of intellectual ability. The inclusive resources and methodology used have made the learning experience accessible to all students. Similarly, the observed homogeneity among students could also be influenced by the adaptation of the questionnaire to an ‘easy-to-read’ format. This inclusive approach facilitates understanding for students with intellectual disabilities (Fajardo *et al*., 2014; Dam *et al*., 2023) and supports their active participation in school health and well-being topics (Vaz *et al*., 2015). Previous health education programmes have adapted their content for people with intellectual disabilities, promoting their autonomy in health-related topics such as pain neuroscience (Osborne & Grimes, 2023), sexual health (Saini & Ahmad, 2021), or oral health (Lai *et al*., 2022).

Regarding the responses on acquired knowledge, students gave high scores to items related to the concepts covered in the educational programme. These results reinforce the effectiveness of the programme in conveying pain neuroscience concepts, consistent with the literature that endorses brief educational interventions to improve knowledge about health issues (Kovacs *et al*., 2011; da Rosa *et al*., 2022). In this regard, our findings are consistent with previous studies, such as that of Louw *et al*. (2018), in which they demonstrated that a brief educational intervention based on PNE can significantly enhance pain knowledge in adolescent students. In another study, these authors observed that this educational approach also modifies erroneous beliefs and promotes appropriate coping strategies (Louw *et al*., 2011). Most previous school-based programmes (e.g., Kovacs *et al*., 2011; Louw *et al*., 2011; Louw *et al*., 2018) were carried out with general student populations without disabilities. Our study adds to this evidence by showing that comparable benefits can also be achieved in inclusive settings, highlighting the feasibility of implementing pain neuroscience education with adolescents who have intellectual disabilities.

However, questions related to the SDGs scored lower compared to the other two constructs evaluated, with moderate mean values, indicating a lower familiarity of students with this topic. This result suggests that students found it challenging to connect the acquired knowledge to global goals such as sustainability. On the other hand, students from rural schools scored higher, with a statistically significant difference compared to students from urban schools. This difference could be attributed to the fact that students in rural settings are more familiar with the SDGs due to agricultural policies and investments in sustainability driven by governmental institutions in rural areas (Leicht *et al*., 2018; Espluga-Trenc *et al*., 2021). Previous studies have shown that education for sustainable development and global citizenship contribute to raising awareness of global issues, such as social justice and climate change, especially in settings where these topics are closer to students’ daily lives (O’Flaherty & Liddy, 2018), reinforcing that rural students may be more exposed to sustainability-related initiatives than their urban peers. In this context, the programme also connects directly with the objectives of the 2030 Agenda, particularly SDG 3 (health and well-being), SDG 4 (quality education), and SDG 10 (reduced inequalities), by promoting accessible health education, fostering inclusive learning environments, and reducing disparities between students with and without intellectual disabilities (General Assembly of the United Nations, 2015).

The construct related to the teacher’s role also scored highly. Previous studies have highlighted that students’ perception of the teacher’s commitment and ability to motivate and convey content has a direct impact on learning outcomes (Wang & Degol, 2015; Hattie, 2009). Consequently, this finding aligns with previous research emphasising the teacher’s role in the effectiveness of health-related educational interventions (Vidal *et al*., 2013; Anyachukwu *et al*., 2024). The item related to the organisation of the activity also received positive scores from students. This result is consistent with that reported by Kovacs *et al*. (2011), who found that a simple and well-organised educational intervention improved adolescent students’ knowledge of back care. Finally, students positively valued the use of pictograms as a didactic resource, as it promotes retention of information and engagement with the content (Anyachukwu *et al*., 2024). These findings suggest that adding PNE to primary education curricula for the promotion of soft skills may be beneficial.

### Clinical implications

This inclusive approach, combined with the structured design of the programme, suggests that it could be used through telehealth approaches, helping to reduce disparities by increasing access to quality pain education in adolescent students with and without intellectual disabilities. Furthermore, given previous evidence supporting classroom-based pain education programme and the high incidence of chronic pain in children, the accessibility of programmes such as this through e-health strategies in rural settings seems a real practical implication.

### Limitations

This study presents some limitations that should be considered when interpreting the results. First, the cross-sectional design of the study does not allow for the evaluation of long-term effects. The findings also rely on self-reported questionnaires, which may introduce response bias. The sample was limited to educational centres in a single region, which may restrict the generalisability of the findings to other populations or educational contexts. Moreover, the absence of long-term follow-up limits conclusions on the sustainability of the observed results. Future studies should include longitudinal follow-ups to assess the impact of the intervention over time, as well as its effect on adolescent students with musculoskeletal pain.

## Conclusion

The hybrid pain neuroscience education programme was associated with high satisfaction and high levels of pain-related knowledge among adolescent students, both with and without intellectual disabilities. The absence of significant differences between ordinary and special education centres, or between rural and urban schools, supports its feasibility as an inclusive and scalable e-health intervention for school-based health promotion. This contribution also reinforces the role of pain neuroscience education within inclusive education and school health, while illustrating its potential to inform digital health promotion strategies.

**Figure 3.**
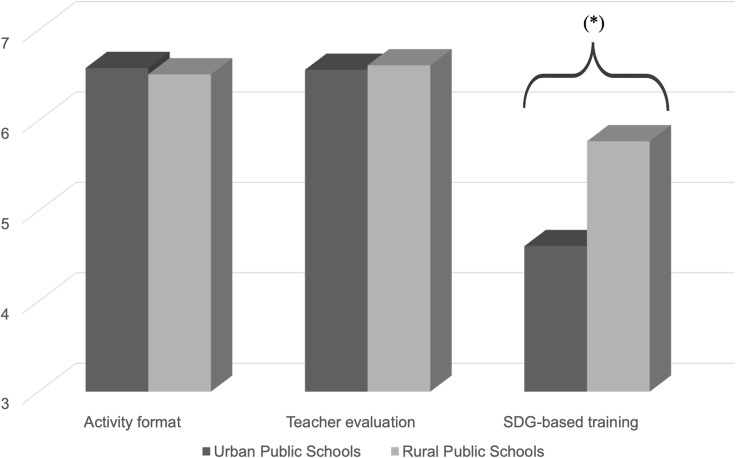
Scores obtained between rural and urban schools. * indicates between-groups statistical significance *p* < 0.001.

**Figure 4.**
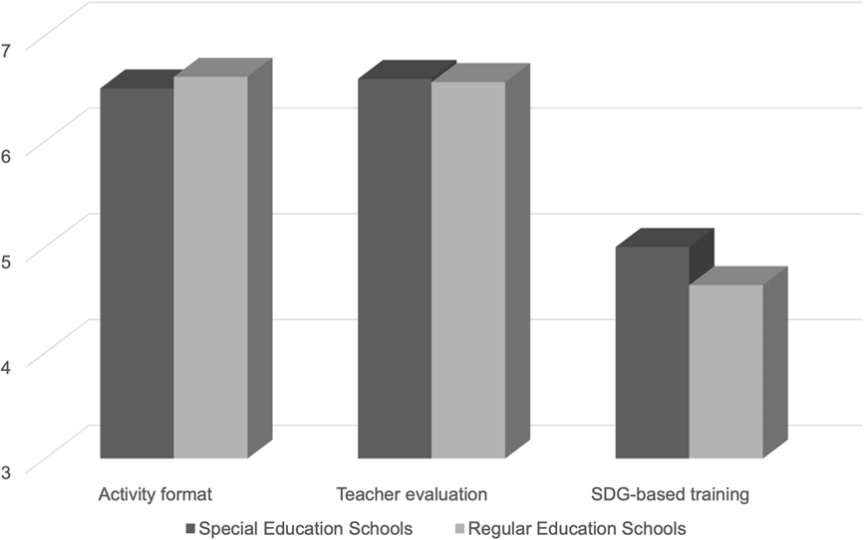
Scores obtained between special and regular education schools.

## Supporting information

Fernández-Morales et al. supplementary material Fernández-Morales et al. supplementary material

## Data Availability

The datasets generated and analysed during the current study are not publicly available due to institutional restrictions but are available from the corresponding author on reasonable request.

## Supplementary material

The supplementary material for this article can be found at https://doi.org/10.1017/neu.2025.10041.
