# Supplementary material for: Pain neuroscience education improves knowledge and satisfaction in adolescents with and without intellectual disabilities: a cross-sectional study
Source: Acta Neuropsychiatr. 2025 Oct 30;38:e2. doi: 10.1017/neu.2025.10041 (PMC13130279; doi:10.1017/neu.2025.10041)
Supplement: Fernández-Morales et al. supplementary material [file S0924270825100410sup001.docx]

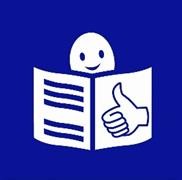


**CUESTIONARIO DE SATISFACCIÓN**

Esta es una encuesta de ***satisfacción** que tienes que rellenar. Antes de rellenar la encuesta, hay que leer cada apartado.

Después, tienes que rellenar la encuesta con los datos que te piden.

Nombre del colegio:

Género del encuestado: Hombre Mujer Otros

Nombre de la asociación: Localidad y provincia donde vives: _ Clase y curso académico:

Para rellenar esta encuesta,

Primero tienes que leer las 15 preguntas.

En segundo lugar, tienes señalar con una X dentro del recuadro en el que aparecen los números.

***Satisfacción:** significa estar contento o a gusto por haber hecho algo.

Cada número tiene una valoración diferente

El número 1 es la puntuación más baja.
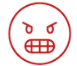


El número 4 es la puntuación media.
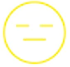


El número 7 es la puntuación más alta.
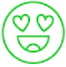


**Estas son las 15 preguntas:**

**1.- Organización.**

¿Qué puntuación le das a la organización de la actividad?


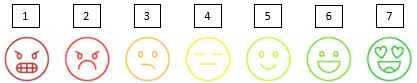


¿Qué puntuación les das a la duración de la actividad?


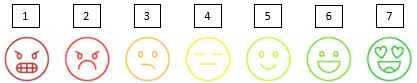


¿Qué puntuación les das al horario de la actividad?


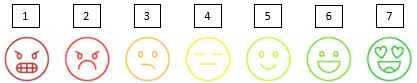


# 2.- Actividad formativa

¿Qué puntuación les das a los conocimientos aprendidos por los alumnos?


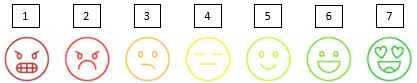


¿Qué puntuación les das a la forma de explicar los objetivos de la campaña?


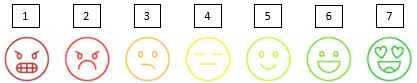


¿Te resultan fáciles los contenidos explicados?


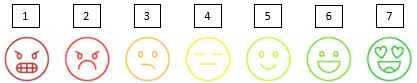


¿Qué puntuación les das a los materiales que se han enseñado como, por ejemplo, la columna vertebral de plástico?


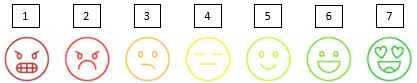


¿Qué puntuación les das a la presentación que has visto en la pantalla, es decir, el Power Point?


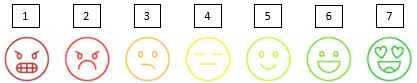


# 3.- Evaluación global

¿Crees que se cumplen los objetivos de la charla?


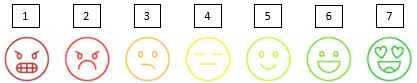


¿Qué opinión en general tienes de la charla?
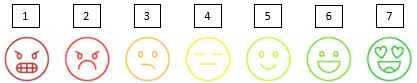


¿Aconsejarías esta actividad a otros centros?


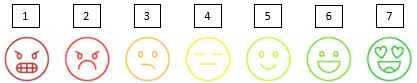


# 4.-Profesor

¿Mantiene el profesor el interés de los alumnos en el taller o aula?


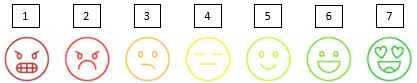


¿Crees que la forma en la que se explican los contenidos tiene buen ritmo?


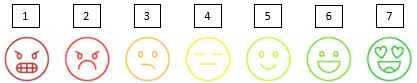


¿Crees que se anima a que los alumnos participen en la charla?


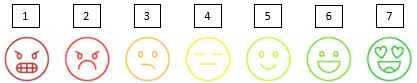


¿Cuál es tu evaluación general del profesor?


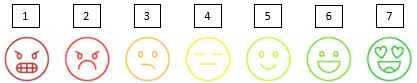


# Estas son otras preguntas que tienes que responder.

Para rellenar estas preguntas,

Primero tienes que leer las 3 preguntas.

En segundo lugar, tienes señalar con una X dentro del recuadro en el que aparecen los números.

Cada número tiene una valoración diferente

El número 1 significa que no estás nada de acuerdo.
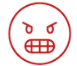
El número 4 significa que estás algo de acuerdo.
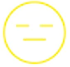


El número 7 significa que estás de acuerdo en todo.
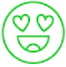


1. ¿Conoces cuáles son los ***Objetivos de Desarrollo Sostenible**?


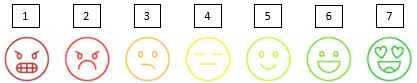


1. ¿Crees que la actividad puede estar relacionada con alguno de los objetivos de desarrollo sostenible?

***Objetivos de desarrollo sostenible:** son herramientas que se usan para proteger el planeta y para que todo el mundo pueda vivir bien ahora y en el futuro.


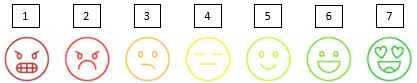


1. ¿Recibes la información sobre los Objetivos de Desarrollo Sostenible por prensa, radio y/o televisión?


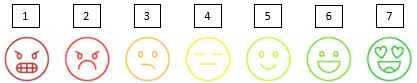


Por último, queremos saber tu opinión sobre la actividad realizada.

Queremos saber tus sugerencias, aportaciones o comentarios que creas que son importantes sobre aspectos como, por ejemplo:

- La organización de la actividad:

....................................................................................................

....................................................................................................

....................................................................................................

....................................................................................................

- La actividad formativa:

....................................................................................................

....................................................................................................

....................................................................................................

....................................................................................................

- Las personas que han dado la charla:

....................................................................................................

....................................................................................................

....................................................................................................

....................................................................................................

Muchas gracias por tu tiempo.


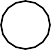

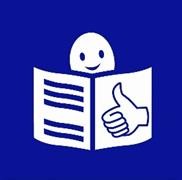


Este documento es un resumen en lectura fácil.

La lectura fácil es una forma de crear documentos que son más fáciles de entender.

Adaptación y validación:

- Aexpainba y FMM
- Marta de Miguel Rodríguez
- Alejandro Moreno Mato
- Cecilio Moreno Mato

c Logo europeo de la lectura fácil.
